# An instrument to assess the education needs of nursing assistants within a palliative approach in residential aged care facilities

**DOI:** 10.1186/s12904-019-0447-0

**Published:** 2019-07-23

**Authors:** Sara Karacsony, Anthony Good, Esther Chang, Amanda Johnson, Michel Edenborough

**Affiliations:** 1 School of Nursing and Midwifery, Western Sydney University, Locked Bag 1797, Penrith, NSW 2751 Australia; 2 School of Nursing, College of Health and Medicine, University of Tasmania, UTAS Education Centre, 1 Leichhardt Street, Darlinghurst, NSW 2010 Australia; 3 School of Nursing, Midwifery and Paramedicine NSW/ACT, Australian Catholic University, PO Box 968, North Sydney, NSW 2059 Australia; 4 School of Social Sciences and Psychology, University of Western Sydney, Locked Bag 1797, Penrith, NSW 2751 Australia

**Keywords:** Nursing assistants, Palliative approach, Residential aged care, Knowledge, Skills, Attitudes, Instrument, Psychometry

## Abstract

**Background:**

Providing quality palliative care in residential aged care facilities (RACFs) (aged care homes) is a high priority for ageing populations worldwide. Older people admitted to these facilities have palliative care needs. Nursing assistants (however termed) are the least qualified staff and provide most of the direct care. They have an important role at the frontline of care spending more time with residents than any other care provider but have been found to lack the necessary knowledge and skills to provide palliative care. The level of competence of this workforce to provide palliative care requires evaluation using a valid and reliable instrument designed for nursing assistants’ level of education and the responsibilities and practices of their role.

**Method:**

The overall study purpose was to develop and test an instrument capable of evaluating the knowledge, skills and attitudes of nursing assistants within a palliative approach in RACFs. Development consisted of a four-phase mixed-methods sequential design. In this paper, the results and key findings following psychometric testing of the instrument in Phase 4 is reported using data collected from a random sample of 17 RACFs and 348 nursing assistants in the Greater Sydney region. Study hypotheses were tested to confirm discriminative validity and establish the utility of the instrument in both research and training assessment.

**Results:**

Individual item properties were analysed for difficulty, discrimination and item-total correlations. Discriminative and structural validity, and internal consistency and test-retest reliability were demonstrated. Three separate questionnaires comprising 40 items were finalised: The Palliative Approach for Nursing Assistants (PANA)_Knowledge Questionnaire (17 items), the PANA_Skills Questionnaire (13 items) and the PANA_Attitudes Questionnaire (10 items).

**Conclusions:**

This study provides preliminary evidence for the validity and reliability of three new questionnaires that demonstrate sensitivity for nursing assistants’ level of education and required knowledge, skills and attitudes for providing a palliative approach. Implications for practice include the development of palliative care competencies through structured education and training across this workforce, and ongoing professional development opportunities for nursing assistants, especially for those with the longest tenure.

**Electronic supplementary material:**

The online version of this article (10.1186/s12904-019-0447-0) contains supplementary material, which is available to authorized users.

## Background

Older people with palliative care needs related to chronic, life-limiting diseases admitted to nursing homes or residential aged care facilities (RACF) as termed in Australia, are highly dependent on skilled, compassionate care. Nursing care is an integral component of residential aged care and is provided by the nursing team comprising registered nurses (RNs), enrolled nurses (ENs) (similar to licensed practical nurses) and nursing assistants (however termed). RACFs were identified over a decade ago as the ‘hospices of the future’ [1] and are now a major provider of aged palliative care as evidenced by the number of older people being admitted to these services for end-of-life care [2–4]. In 2015, 34% of all Australian deaths occurred within RACFs with more than 60% of residents dying within six months of admission [5]. The majority of permanent residents assessed as requiring palliative care were in the 85 years and older age group and over half of all residents in RACFs have a diagnosis of dementia [5, 6].

Whether palliative care is required on admission to the RACF or at some later stage, nursing assistants are involved in providing comfort care and in the resident’s transition to end of life. A palliative approach is considered best practice for this population with its focus on the needs of the person, not the disease [7, 8] and can be delivered by a range of non-specialists at point of admission to the service [9]. Nursing assistants who provide routine personal care such as bathing, dressing or grooming, and assisting elderly, convalescent or disabled people with eating, mobility and communication provide most direct care to residents and spend more time with them than any other care providers [10]. Nursing assistants are required to work under the supervision of RNs or ENs who are responsible for the overall care of residents. However, recruitment has shifted to nursing assistants as the mainstay of the RACF workforce (70%) [11], and there are fewer RNs occupying a supervisory role [12].

Although nursing assistants are best placed to identify and support residents’ physical, psychological, emotional and spiritual needs [13], and provide support to family carers reflecting core elements of palliative care, they require palliative care knowledge, essential skills and positive attitudes necessary for adopting core values of palliative care, such as an open attitude to dying and death [7, 14, 15]. Of the nursing assistants (*n* = 108,126) surveyed in the Australian National Aged Care Workforce Census and Survey 2016, only 7.4% reported a specialised qualification in palliative care and 72% identified this as a priority area for training [11]. Nursing assistants are less likely than RNs or ENs to attend non-mandatory professional development education [16] and this may not be optimally aligned to the demands of the workplace [17]. Furthermore, the education curricula of nursing assistants, in general, has been found to vary in content [17] and there is no specific prior learning or mandatory requirement in Australia to participate in pre-service education nor is there currently any licensing, legislative, or regulatory requirements attached to the entry-level industry qualification [18]. With respect to palliative care, the only educational unit that explicitly addressed a palliative approach to care [19] was changed in 2015 from a core unit to an elective within this Australian qualification [18]. This means that nursing assistants, as a broad-based group, may not have any pre-service education in palliative care.

It is important to be able to evaluate nursing assistants’ palliative care knowledge, skills and attitudes with a validated instrument. When nursing assistants have been evaluated with existing instruments, they have been found to have a low knowledge of palliative care [20–25] and deficits in skills and attitudes [21, 26]. However, only one instrument out of seven identified and critically examined was developed for the nursing assistants’ role limiting the validity of study findings related to this group [27].

This study has developed an instrument designed to evaluate the knowledge, skills and attitudes of nursing assistants within a palliative approach in order to elicit the educational needs relative to their role and responsibilities in providing care with a palliative approach. This paper reports the results of Phase 4 of a four-phase mixed methods study to validate three questionnaires entitled PANA (Palliative Approach for Nursing Assistants) and discusses the main findings of this phase of the study (Additional file 2).

To test the validity of the instrument for nursing assistants, it was hypothesised that an instrument designed specifically for nursing assistants’ level of education and scope of practice would:

I. Perform better than the Palliative Care Quiz for Nursing (PCQN) [28] in discriminating knowledge of a palliative approach between groups of nursing assistants.
II. Demonstrate that experience measured as length of time in the nursing assistants’ role will be a better predictor than education in discriminating knowledge of a palliative approach between groups of nursing assistants.
III. Detect differences between groups of nursing assistants for knowledge of a palliative approach based on experience in the role.
IV. Detect differences between groups of nursing assistants in self-perceived skills for a palliative approach based on experience in the role.
V. Detect differences between groups of nursing assistants for attitudes towards a palliative approach based on experience in the role.

The relevance of these hypotheses to the development of the instrument is, first, to confirm that the instrument can be used to evaluate differences in scores between groups with varying levels of experience and education; and, second, to provide insight into the effectiveness of educational processes.

The broader study was conducted over four sequential phases according to recommended psychometric processes for instrument development [29, 30]. The four phases were item generation, content validation with experts, pilot testing and field testing and are shown in summary in Table 1. Human Research Ethics approval was obtained from Western Sydney University (H9963).

**Table 1:** Summary of the four sequential phases of the study

Phases	Data collection	Analysis/Products
Phase 1 Item Generation	Semi-structured interviews with nursing assistants (*n* = 25)	Transcribed texts Themes – categories – item pool: 51 knowledge items, 48 skill items and 36 attitudes items.
Phase 2 Instrument Development	Survey method Four groups of experts (*n* = 9–12): academics in the field of palliative/aged care; industry representatives with responsibility for training and development; RNs supervising the direct care provided by nursing assistants in RACFs; and, nursing assistants with a Certificate IV in Aged Care and/or at least five years’ experience in their role.	Content Validity Index (CVI) two rounds and one face validation; CVI value for items rated on a four-point ordinal scale: 1 = not clear, not relevant 2 = not quite clear, not quite relevant (requires major revision); 3 = clear, relevant (with minor revision); 4 very clear, very relevant Draft questionnaires within one instrument titled PANA (Palliative Approach for Nursing Assistants) (85 items) Dichotomous/scaled variables
Phase 3 Pilot Testing	Survey method Two RACFs, purposive sampling (*n* = 61) Inclusion: Group 1: less than or equal to two years’ experience in role; Group 2: between two and five years’ experience in role; Group 3: more than five years’ experience in role	Descriptive statistics, mean scores, standard deviations, confidence intervals, summary tables Refinements of items and response options
Phase 4 Instrument Testing	Survey method 17 RACFs, random sample (*n* = 348) Inclusion: Group 1: less than or equal to two years’ experience in role; Group 2: between two and five years’ experience in role; Group 3: more than five years’ experience in role	Descriptive statistics, Individual item analysis, mean scores, Kendall’s Tau Correlation, analysis of variance, factor analysis, Cronbach’s Alpha, Pearson’s product moment correlation coefficient, Final instrument: PANA_Knowledge Questionnaire; PANA_Skills Questionnaire; PANA_Attitudes Questionnaire

### Instrument development process

Phase 1 commenced with the generation of items. The *Guidelines for a palliative approach in residential aged care* [7] (Additional file 1) provided the conceptual framework for the inclusion of content in the instrument (included as supplementary material). The educational unit *Deliver care services using a palliative approach* [19], offered in the nationally recognised aged care qualifications [18, 31], was used to delineate content specific to the performance criteria or ‘scope of practice’ of nursing assistants.

Phase 2 involved content validation by experts. Clarity and relevance were evaluated using the Content Validity Index (CVI) on 135 items [32]. Of the 89 items retained following the content validation process, content validation of these total items in the new instrument was assessed. First, the average of all items rated either 4 or 3 were summed to produce an overall score for content validity of 0.96, and then the S-CVI universal method, which requires agreement by all experts, was applied. Eighty-five items were endorsed, giving an overall score for the instrument’s content validity of 0.99.

In Phase 3, a final pool of 85 items was established for pilot testing, consisting of 28 knowledge, 38 skills, and 19 attitude items. The prefix PANA (Palliative Approach for Nursing Assistants) was adopted for each of the three questionnaires. The procedure and results of this phase have been reported elsewhere [33]. As a result of pilot testing in Phase 3, amendments were made to two knowledge questions and the response options for the skills statements. The result was an 85-item instrument ready for field testing and psychometric evaluation.

## Methods

### Phase 4 Field testing procedure

#### Validity analysis

Three types of measurement-related validity were examined. Known groups (discriminative validity), convergent and divergent validity, and structural (construct) validity using an exploratory factor analysis was assessed. For the known groups, nursing assistants were allocated into one of three groups at time of completion of the questionnaires based on information provided in the demographic questions. The three groups were: group 1: staff with less than or equal to two years’ experience in role; group 2: between two and five years’ experience in role; and, group 3: staff with more than five years’ experience in role.

#### Reliability analysis

Internal consistency was assessed with Cronbach’s alpha coefficient [34] and stability (test-retest reliability) was assessed with an intra-class correlation (ICC), which is sensitive both to agreement of scores and to association between scores [35].

##### Sample

A manual method of random allocation was used to recruit facilities for Phase 4 field testing. The Aged Care Service List New South Wales (NSW) (June 2014), which provides information on aged care services subsidised by the Australian Government, was used to identify facilities within the Greater Sydney area with more than 50 operational places or ‘beds’ (*n* = 116). These facilities were allocated a number corresponding to the List. One person randomly selected 20 numbers representing these eligible facilities from a concealed container in front of the study team. Twenty facilities were sought for recruitment on the basis that if each facility only had a minimum of 50 places or ‘beds’, the mean ratio (worker: resident) of 0.7 direct care workers, with the majority of these being nursing assistants (70%) [11], there would be a sample of 490 (20x50x0.7x0.7) nursing assistants. A minimum sample of 300 participants was sought based on statistical advice and published literature on sample size required for exploratory factor analysis [36–38]. Of these twenty facilities, six initially declined to participate: two of these were later granted organisational permission and a randomly selected back-up site using the same procedure that determined the initial sites agreed to participate. Altogether, 17 sites were included, with 1888 operational places yielding a potential sample of 1321 nursing assistants. Approval was obtained from each participating facility or from the organisation’s research department if the facility was part of a larger organisation.

### Participants

The number (or approximate number) of nursing assistants working in each facility was provided by the Facility Care Manager (FCM). All nursing assistants providing direct care to residents from the participating RACFs were eligible to participate. The FCM or delegated other was responsible for distributing the instrument booklet to interested staff. A subsample of thirty nursing assistants was also sought to complete the instruments a second time for the purpose of test-retest reliability and responsiveness.

### Instrument booklet

A 16-page A4 instrument booklet was compiled in English containing the three new questionnaires developed and refined in Phases 1, 2 and 3, and the Palliative Care Quiz for Nurses (PCQN) [28]. The PCQN was designed to test nurses’ knowledge of palliative care and has been used in a number of Australian studies to test nursing assistants’ knowledge of palliative care [20, 23, 39]. Participant information, agreement to participate and demographic items were presented at the beginning of the booklet. Demographic data related to gender, age, whether born in Australia, years of experience in role and highest level of education were collected. For each instrument, instructions and generic information, contact details of the researcher, preceded its set of items. To ensure participant confidentiality, separate survey administration instructions and an individual envelope stamped ‘Confidential’ were provided for each paper copy. A link on the paper-based administration instructions provided the address to the online version of the questionnaires. Consent was implicit when participants indicated their agreement to proceed following the participant information. Whether participants completed the questionnaires in their own time or in work time was left to the discretion of the individual facilities and no assistance was given by RACF or research staff for completion.

#### PANA_Knowledge questionnaire

This questionnaire consisted of 28 items testing nursing assistants’ knowledge of a palliative approach. Response options to each knowledge item were True/False/Don’t Know with each correct item scored one and incorrect or DK responses scored zero with a possible total correct score of 28.

#### PANA_Skills questionnaire

The second questionnaire comprised 38 skill items, the purpose of which was to identify nursing assistants’ self-perceived skills when providing care with a palliative approach. The response options were: I know how to do this; I am unsure how to do this; I do not know how to do this. Each know how response was scored one and unsure/do not know how responses were scored zero with a total possible response of 38.

#### PANA_Attitudes questionnaire

The third questionnaire comprised 19 attitudinal statements, the purpose of which was to understand how nursing assistants feel about providing care with a palliative approach. A five-point Likert scale was used to collect responses, a number shown to be both reliable and low enough for participants to answer quickly [40]. Responses options were: Strongly Agree (SA); Agree (A); Unsure (U); Disagree (D); Strongly Disagree (SD). Items scored SA/A reflecting more positive responses were scored one point, items scored U, D, SD were scored zero with a total positive score of 19.

#### Palliative care quiz for nurses (PCQN)

The fourth and last instrument was the PCQN which is a 20-item quiz. Response option in the quiz were True/False/Don’t Know with each correct item scored one and incorrect or DK responses scored zero with a possible total correct score of 20.

### Analysis

Analyses of demographic data was conducted in Excel and all other analyses using the Statistical Package for the Social Sciences (SPSS) version 24 [41]. Analysis evaluated individual item properties for item difficulty, item discrimination, item-total correlation above 0.20 [42] and mean group scores.

#### Individual item analysis

Item difficulty and item discrimination provide important information about the performance capabilities of items to differentiate levels of the attributes being tested across the target population [43, 44]. Item discrimination and item difficulty indices are used to measure the reliability of scores and are independent of one another: an item can be easy or difficult whether it is discriminating or non-discriminating [43]. Difficulty and discrimination indices provide the criteria for item inclusion in the definitive version of the new knowledge instrument. Item difficulty analysis was not applied to the attitudinal items on the basis that attitudes are neither correct nor incorrect; nor was it applied to the self-reported skills items.

A random half of the sample (*n* = 174) was used for item discrimination and item difficulty, using the top 25% of scorers (*n* = 43) and the bottom 25% of scorers (n = 43). The number of correct items for each set was calculated, and for each item, the percentage of participants in each group who answered correctly was obtained. The two percentages (high scorers minus low scorers) were then subtracted from each other to arrive at the item’s discrimination index. The decision to retain an item was based on a positive discrimination index above .20 [28, 45, 46]. The item difficulty index provided the percentage of participants in the half-sample who scored each item correctly. Items that are answered either correctly or incorrectly by a high percentage of participants (e.g. 95% or more do not allow any discrimination among the users of the instrument and are candidates for deletion [36]. The values used in this study were 0.10 and 0.90.

## Results

### Participants

Nursing assistants (*n* = 353) across 17 sites completed the demographic questions (Table 2). Of these, 348 also completed the knowledge questions; 343 completed demographic, knowledge, skill and attitude items, and 326 completed all questions including the PCQN. The majority was between 25 and 34 years of age and held the Certificate III in Aged Care. Forty-one per cent (*n* = 145) had been employed in their role for more than five years in the role. For those with the least experience in group 1, only 11.3% were born in Australia compared to 53% of staff in group 3. Role descriptions included Assistant in Nursing (AIN), care companion, care staff, aged care worker, care services employee (CSE), personal carer/care assistant. Five participants did not proceed to the first questionnaire. The reasons for staff not progressing beyond the demographic questions are not known.

**Table 2:** Demographic characteristics of sample Phase 4 (*N* = 353)

Variable	Group 1 (less than two years’ experience)	%	Group 2 (between two and five years’ experience) (*n* = 119)	%	Group 3 (more than five years’ experience) (*n* = 145)	%
Total	89	25.2	119	33.7	145	41.1
Gender						
Female	75	84.3	101	84.9	128	88.3
Male	14	15.7	18	15.1	17	11.7
Age mean (years) [SD]	30 [8.36]^a^		38 [10.87]^b^		49 [11.18]^c^	
Years range	21–59		23–64		24–70	
Australian-born		11.3		35.5		53.2
Highest education						
Year 10 or equivalent	4	4.5	2	1.7	9	6.2
Year 12 or equivalent	9	10.1	8	6.7	6	4.1
Cert. 3 Aged Care	48	53.9	47	34.1	43	29.7
Cert. 4 Aged Care	11	12.4	26	21.8	57	39.3
Diploma or Certificate TAFE	11	9.0	15	12.6	20	13.8
Undergraduate nursing	9	10.1	21	17.6	10	6.9

^a^missing data =5

^b^missing data =6

^c^missing data =5

#### PANA_Knowledge questionnaire

The results of the Item Discrimination Index and the Item Difficulty Index are presented in Table 3.

**Table 3:** Item characteristics PANA_knowledge questionnaire

No.	Item	Discrimination Index	Difficulty Index	Item-total correlation	Alpha if item removed	Final alpha
1.	A palliative approach aims to improve quality of life when people have an illness or a condition that affects how long they will live.*	23	.87	.40	0.79	0.72
2.	A palliative approach supports comfort but does not provide a cure.*	33	.87	.41	0.79	0.73
3.	A palliative approach may be required for some people for months or years while for others it may be required for hours or days.*	21	.92*	.39	0.79	0.72
4.	The needs of people requiring a palliative approach are the same.	39	.67	.37	0.79	0.73
5.	A palliative approach is offered when treatment will not help the person to live longer.*	41	.79	.40	0.78	0.72
6.	People who have advanced cancer, severe lung or heart or kidney disease or advanced dementia benefit from a palliative approach. *	44	.74	.39	0.78	0.72
10.	Families can often experience grief before the death of their family member. *	16	.90*	.37	0.79	0.73
11.	It is better to provide information about a palliative approach to people from culturally and linguistically diverse backgrounds in English.	27	.29	.27	0.79	0.74
12.	The reason why a person receives nutrition through a Percutaneous Gastrostomy (PEG) tube (a feeding tube into the stomach) is because he/she can no longer swallow safely. *	25	.89	.40	0.79	0.72
13.	Identifying symptoms (physical signs) is the first step in being able to manage symptoms. *	20	.85	.39	0.79	0.72
14.	Pain relief before providing physical care, such as dressing a wound, can help a person experiencing pain feel more comfortable. *	30	.87	.39	0.78	0.72
15.	When a person is receiving pain relief, they no longer feel pain.	39	.75	.31	0.79	0.73
18.	Families or carers who know the person best are usually the first to detect changes in a person’s condition. *	19	.91*	.38	0.78	0.73
19.	A person expressing a wish to die means that the person will die soon.	27	.83	.21	0.79	0.74
24.	Bladder and bowel problems can cause discomfort when a person approaches the end of life.*	25	.88	.29	0.79	0.71
27.	When a person has experienced a deterioration over time, it is a sign that they are approaching the end stage of their illness.*	37	.65	.19	0.80	0.75
28.	Signs that death is near can be present hours to days before death occurs.*	33	.71	.24	0.79	0.74

* True

For the PANA_Knowledge Questionnaire, high scorers (top 25%) answered between 24 and 27 items correctly (M = 25, SD = 0.67); low scorers (bottom 25%) answered between 8 and 20 items correctly (M = 18.2, SD = 2.77). Twelve items were candidates for deletion, based on values below 0.10 and above 0.90 for difficulty and a negative discrimination index below 0.20 and had item-total correlations less than 0.20. One item (22) (Spiritual care identifies what is important to a person) scored above 0.10 and less than 0.90 for difficulty, the low item-total correlation made it too a candidate for deletion.

For the 16 knowledge items retained following the individual item analyses, Cronbach’s alpha was 0.69 (reduced from 0.79 on the original 28 items). To ensure alpha did not drop below the acceptable lower limit of α = 0.70 [44, 47], two items falling below 0.20 (0.16, 0.19) in discrimination were retained as both these items had scores below 0.90 for difficulty among the three groups of nursing assistants. Retaining these items increased alpha to 0.72 and enhanced content coverage. The removal of item 22 further improved Cronbach’s alpha from α = 0.72 to α = 0.74. A total of 17 items were retained as the final set for the PANA_Knowledge Questionnaire, shown in Table 3.

Descriptive statistics for the final knowledge items are shown in Table 4.

**Table 4:** Descriptive statistics for final knowledge items

Groups	Mean	N	Std. Deviation	Minimum	Maximum	Range
1	13.49	88	2.501	4	17	13
2	13.52	116	2.455	3	17	14
3	14.03	144	1.918	8	17	9
Total	13.72	348	2.269	3	17	14

#### PANA_Skills questionnaire

The results of the item discrimination, item-total correlation and alpha values for skills items are presented in Table 5. Twenty-five items were candidates for deletion based on a discrimination index below .20. The thirteen retained items correlated above 0.30. Cronbach’s alpha for individual items if the item was removed from the scale was between 0.79 and 0.81, with the final alpha on the remaining 13 items equal to 0.81. A total of 13 items were retained as the final item set for the PANA_Skills Questionnaire.

**Table 5:** Item characteristics PANA_Skills Questionnaire

Item no.	Item	Discrimination Index %	Item-total correlation	Alpha if item removed	Final Alpha
1	Observe what a person can do without assistance.	21	.44	.93	.80
3	Assist in updating care plans.	47	.34	.93	.81
9	Direct families to other members of the care team when they need further advice.	42	.47	.93	.80
13	Care for a person with challenging behaviours.	26	.52	.93	.80
14	Raise the concerns of (advocate for) the individuals in my care.	30	.44	.93	.80
16	Observe for pain using a valid and reliable pain assessment tool.	63	.41	.93	.79
18	Provide non-medication strategies, such as gentle massage, in order to manage pain.	49	.48	.93	.79
19	Evaluate the effectiveness of pain management strategies using a validated pain assessment tool.	77	.41	.93	.79
27	Contribute to problem solving to seek solutions.	28	.43	.93	.80
29	Recognise the signs when an individual is in the last days or hours of life.	42	.45	.93	.80
31	Attend to a dying individual’s care.	23	.50	.93	.80
33	Find ways to cope with my own emotional responses when a person I have been caring for has died.	21	.51	.93	.80
35	Reflect on what I say and do when providing a palliative approach.	23	.57	.93	.80

Descriptive statistics for the final skills items are shown in Table 6.

**Table 6:** Descriptive statistics for final skills items

Groups	Mean	N	Std. Deviation	Minimum	Maximum	Range
1	10.80	87	2.587	2	13	11
2	10.96	115	2.194	3	13	10
3	11.38	141	2.160	4	13	9
Total	11.09	343	2.294	2	13	11

#### PANA_Attitudes questionnaire

The results of item discrimination, item-total correlation and alpha values for attitudinal items are presented in Table 7. Twelve items were candidates for deletion based on a discrimination index below 0.20. Three items showed a value below 0.20 for the item-total correlation, two of these were reverse-scored items and showed negative values. With only seven items above 0.20 for discrimination, Cronbach’s alpha dropped from 0.77 on the original 19 items to 0.36 on the revised sample of seven items. To ensure an acceptable alpha (0.70–0.80), six items were retained using 0.10 as the cut-off. However, three of these items had low item-total correlations and these were excluded. Cronbach’s alpha for the ten retained items was 0.80 and all item-total correlations were above 0.10. These 10 items were the final item set for the PANA_Attitudes Questionnaire.

**Table 7:** Item characteristics PANA_Attitudes Questionnaire

no.	Item	Discrimination Index %	Item-total correlation	Alpha if item removed	Final Alpha
1.	A palliative approach can help a person’s quality of life.	17	.47	.76	.78
4.	Being aware of a person’s emotional, social and spiritual needs is my responsibility.	28	.39	.76	.79
6.	Caring for a person with a palliative approach is rewarding.	14	.40	.76	.78
7.	Providing a palliative approach based on an individual’s wishes improves quality of life.	19	.45	.76	.78
10.	Understanding physical and emotional changes at the end of life helps me provide care with a palliative approach.	12	.65	.75	.77
11.	I feel comfortable when an individual receiving a palliative approach says they are ready to die.	70	.28	.77	.81
13.	I make a difference to a person’s day when I provide care with a palliative approach.	21	.65	.75	.76
14.	I have an important role to play in pain assessment and management.	33	.52	.75	.78
16.	When I provide care with a palliative approach, I think about the whole person.	17	.61	.75	.77
17.	Privately sharing experiences with colleagues is important when providing a palliative approach.	49	.40	.76	.79

Descriptive statistics for the final attitudes items are shown in Table 8.

**Table 8:** Descriptive statistics for final attitudes items

Groups	Mean	N	Std. Deviation	Minimum	Maximum	Range
1	8.47	85	1.666	0	10	10
2	8.54	116	1.696	1	10	9
3	9.05	142	1.251	2	10	8
Total	8.73	343	1.538	0	10	10

### Validity

#### Discriminative validity

To evaluate the discriminative capabilities of the PANA_Knowledge Questionnaire compared to the existing PCQN, a between-groups analysis and test of significance using ANOVA was performed. A two-way ANOVA was performed on the final 17 items of the PANA_Knowledge Questionnaires with level of experience as the first factor and level of education as the second factor (Hypothesis I and II). The results are presented in Table 9. Results trend towards a correlation between higher knowledge scores and experience in role as measured by the PANA_Knowledge Questionnaire (F = 2,390, df = 2, ρ = 0.093); this is not the case for level of education or for the interaction of education and experience on total knowledge scores (F = .853, df = 10, ρ = 0.578).

**Table 9:** Two-way ANOVA for knowledge scores: experience in role and level of education

	Tests of Between-Subjects Effects				
	Dependent Variable: Total Knowledge				
	Type III Sum of Squares	df	Mean Square	F	Sig.
Corrected Model	98.343^a^	17	5.785	1.131	.322
Intercept	29813.909	1	29813.909	5829.862	.000
@What is the highest level of education you have completed	43.978	5	8.796	1.720	.129
@Please indicate how long you have been working in this role	24.440	2	12.220	2.390	.093
@What is the highest level of education you have completed* @Please indicate how long you have been working in this role	43.627	10	4.363	.853	.578
Error	1687.620	330	5.114		
Total	67305.000	348			
Corrected Total	1785.963	347			

Note: a. R Squared = .055 (Adjusted R Squared = .066)

A two-way ANOVA was also performed on the PCQN to compare the instrument’s performance with the PANA_Knowledge Questionnaire. The results showed no significant difference between groups of nursing assistants based on years or experience or level of education or the interaction of experience and education on total knowledge scores using the PCQN (see Table 10).

**Table 10:** Two-way ANOVA for PCQN scores: experience in role and level of education

Tests of Between-Subjects Effects					
	Type III Sum of Squares	df	Mean Square	F	Sig.
Corrected Model	211.177^a^	17	12.422	1.174	.284
Intercept	6285.356	1	6285.356	594.054	.000
@7. Please indicate how long you have been working in the role_A	7.910	2	3.955	.374	.688
@5. What is the highest level of education you have completed_A	50.124	5	10.025	.947	.450
Error	3258.777	308	10.580		
Total	17689.00	326			
Corrected Total	3469.954	325			

Note: a. R Squared = .061 (Adjusted R Squared = .009)

A one-way ANOVA was performed on the PANA_Skills questionnaire and the PANA_Attitudes Questionnaire (hypotheses IV and V); the results are presented in Tables 11 and 12 and show that there was no significant difference between groups based on experience for skills scores. However, there was a significant difference between groups for the attitudes scores, demonstrating that the PANA_Attitudes Questionnaire was able to discriminate between groups based on experience (F = 5.252, df = 2, ρ = 0.006).

**Table 11:** One-way ANOVA for skills scores and experience in role

Total Skills					
	Sum of Squares	df	Mean Square	F	Sig.
Between Groups	21.235	2	10.617	2.031	.133
Within Groups	1777.780	340	5.229		
Total	1799.015	342

**Table 12:** One-way ANOVA for attitude scores and experience in role

Total Attitudes					
	Sum of Squares	df	Mean Square	F	Sig.
Between Groups	24.241	2	12.121	5.252	.006
Within Group	784.616	340	2.308		
Total	808.857	342			

#### Divergent validity

The prediction that there would be a divergent relationship between the PANA_Knowledge Questionnaire and the PCQN was supported by the results presented in Table 13.

**Table 13:** Correlation between scores PANA_Knowledge Questionnaire and PCQN

			Score PANA_Knowledge Questionnaire	Score PCQN
Kendall’s tau_b	Score PANA_Knowledge Questionnaire	Correlation Coefficient	1.000	.166
		Sig. (1-tailed)	–	.000
		N	348	343
	Score PCQN	Correlation Coefficient	.166	1.000
		Sig. (1-tailed)	.000	–
		N	348	343

A correlation coefficient using Kendall’s tau statistic specifying a one-tailed test (the hypothesis was directional) was performed on the PANA_Knowledge Questionnaire to establish whether there was convergence or divergence with the PCQN. A small correlation (r = .166) was observed between scores on the PANA_Knowledge Questionnaire and those on the PCQN, with a significance value of less than 0 .001 (ρ < 0.001). This value indicates that the probability of getting a correlation coefficient of this magnitude in the sample of 350 participants is very low and that there is little correlation between the two knowledge instruments based on this sample.

##### Structural validity: exploratory factor analysis

A principal component analysis was conducted on the full final item set of knowledge, skill and attitudes. The Kaiser-Meyer-Olkin Measure of Sampling Adequacy verified the sample’s adequacy for the analysis with a value close to 1.0 (KMO .903). The Bartlett’s test of sphericity, Approximate Chi-Square was 2435.617 and was significant (ρ. > 001) indicating that correlations between individual variables (items) were sufficiently different from one another and suitable for factor analysis [37]. As well, the correlation matrix was examined to identify that there were correlations between variables > 0.3 and that the data set was factorable. Components with loadings above 0.3 were extracted. A pattern and structure matrix was generated for interpretation. The analysis reduces variables into clusters and provides conceptual representation of the data. This analysis showed that the unrotated first principal component accounted for only 14.2% of the variance. Following oblique rotation, 13 components had eigenvalues over Kaiser’s criterion of 1, and in combination explained 58.7% of the variance. The scree plot and a parallel analysis confirmed five components that accounted for only 36% of the variance, whereas a minimum variance of 50% is recommended [44]. This low variance as well as the minimal variance in the correlations of the knowledge items with all other variables, the inclusion of mixed variables and problems with equivalence between variables resulted in the retention of the knowledge and skills items as separate indices and not as separately scored subscales within a single instrument [48] .

The PANA_Attitudes Questionnaire with response options provided as ordinal variables was independently subjected to a principal components analysis with oblique rotation. Analysis was conducted on a sample of 343 following the exclusion of five cases with missing values. The Kaiser-Meyer-Olkin Measure of sampling adequacy was .861 and the Bartlett’s Test of Sphericity was significant (ρ < 0.001) indicating that the data was factorable. The variables in the anti-image correlation ranged from .782 to .927. Two components made up the subscales. The pattern matrix was used for interpretation as follows: Component 1 represented holistic care; Component 2 represented a palliative approach. Table 14 attached presents the rotated component loadings, communalities, eigenvalues, percentage of variance, and alpha values.

**Table 14:** Factor structure PANA_attitudes questionnaire

	Attitude items	Components		
		1	2	h^2^
Component 1	Holistic care			
16	When I provide care with a palliative approach, I think about the whole person	0.777	−.025	0.608
14	I have an important role to play in pain assessment and management	0.749	−.074	0.577
10	Understanding physical and emotional changes at the end of life helps me provide care with a palliative approach	0.699	.127	0.619
13	I make a difference to a person’s day when I provide care with a palliative approach	0.644	0.245	0.610
11	I feel comfortable when an individual receiving a palliative approach says they are ready to die	0.570	−.137	0.741
17	Privately sharing experiences with colleagues is important when providing a palliative approach	0.508	−.097	0.530
4	Being aware of a person’s emotional, social and spiritual needs is my responsibility	0.503	.069	0.323
Component 2	A palliative approach			
7	Providing a palliative approach based on an individual’s wishes improves quality of life	−.075	0.904	
1	A palliative approach can help a person’s quality of life	.078	0.768	
6	Caring for a person with a palliative approach is rewarding	.328	0.417	
	Eigenvalues	3.95	1.04	
	% of variance	39.475	10.365	
	Alpha	0.75	0.66	

Note: The overall Cronbach’s alpha for the PANA_Attitudes Questionnaire was 0.80 with 0.75 and 0.66 for each subscale. Items above 0.40 were specified [37]

### Reliability

Internal reliability was assessed with Cronbach’s alpha coefficient and test-retest reliability was assessed with intra class correlations (ICC). This was performed on all original items (*n* = 85) as well as on each separate item set using a two-way random effects model with a single measure and consistency of scores. The results are presented in Table 15. Twenty participants completed the three questionnaires of the instrument at time 1; only 16 completed the instrument at time 2, which was a small sample. One participant did not proceed to the PANA_Attitudes Questionnaire at time 2.

**Table 15:** Intraclass correlation, Cronbach’s alpha, 95% confidence intervals and significance for PANA_KSAq, PANA_Knowledge Questionnaire, PANA_Skills Questionnaire, PANA_Attitudes Questionnaire

Instrument	Sample	Test-retest ICC	Cronbach’s alpha	95% CI [range]	Sig.
PANA_KSAq (n = 85)	16	0.546	0.706	[0.067, 0.820]	.014
PANA_Knowledge Questionnaire (n = 17)	16	0.709	0.830	[0.344, 0.888]	.001
PANA_Skills Questionnaire (*n* = 13)	16	0.601	0.751	[0.167, 0.840]	.005
PANA_Attitudes Questionnaire (n = 10)	15	0.335	0.502	[−.195, 0.713]	.102

## Discussion

The psychometric properties of the new instrument were evaluated to establish a final set of items that meet recommended criteria for validity and reliability. An analysis of the PANA_Knowledge Questionnaire’s psychometric properties indicates it has good internal consistency (0.74) and retest reliability (0.709) performed better than the PCQN in discriminating knowledge of a palliative approach between more and less experienced staff trending towards a correlation with higher knowledge scores and experience in role.

The 13-item PANA_Skills Questionnaire is an index of overall self-perceived skill in the delivery of a palliative approach. The instrument indicates high internal consistency (0.81) and adequate stability (0.608). The 10-item PANA_Attitudes Questionnaire, scored on a 5-point ordinal scale demonstrates strong discriminative validity (*p* = 0.006) demonstrated by its ability to detect a statistical difference between groups of nursing assistants based on experience in the role, and high internal consistency (0.80). The ICC value for the instrument is 0.335. This low value may reflect factors such as workplace satisfaction/dissatisfaction or changes in the emotional state of the participant [49], as well as the small sample completing the retest (*n* = 15).

Other key findings that provide insight for future workforce development emerged from these results. Firstly, nursing assistants demonstrated higher scores on the new PANA_Knowledge Questionnaire compared to scores on the PCQN [28], consistent with other studies reporting knowledge results for nursing assistants using this instrument or elements of it [20, 21, 23, 25]. Similarly, nursing assistants, when tested with the Palliative Care Survey (PCS) [22], have demonstrated low scores compared to registered or licensed nurses [24, 50]. While it is expected that these staff will perform better than nursing assistants considering educational differences related to biomedical and pharmacological knowledge, and health literacy, the instruments were not developed for nursing assistants and include elements that are not usual practice for nursing assistants. By comparison, the PANA Knowledge items satisfactorily tap into the knowledge required for the nursing assistant’s role and demonstrate a range of knowledge scores from low to high (M = 13.72 out of a possible 17 correct responses in the final instrument). In contrast to previous studies that have shown that nursing assistants have low overall knowledge of palliative care [20–24], this study shows that across the spectrum of experience, nursing assistants perform well when measured with a tailored instrument developed using the target group in the development process. A desirable quality of a knowledge instrument is that it can discriminate between participants with specific characteristics pertaining to that knowledge [28] as the PANA_Knowledge Questionnaire is able to demonstrate according to experience.

The second major finding is that there was no significant difference in nursing assistants’ skills across level of experience when evaluated with the new PANA_Skills Questionnaire. These findings are within the context of a relatively stable Australian RACF workforce and relatively long tenure for many staff with 34% having stayed in the same job for between one and four years and 28% between four and nine years [11]. A major implication in the lack of difference in skills scores between less and more experienced staff is that the skills of nursing assistants, across years of experience, remain largely static when measured with a tailored instrument. To date, the scope of practice of nursing assistants is not clearly defined, and there are no standards or regulatory framework for this workforce. Usual activities delegated to nursing assistants relate to personal care and activities of daily living in support of registered and enrolled nurses. Uncertainty regarding the role and scope of nursing assistants and the delegation of additional activities beyond usual practices have been identified as a result of role ambiguity, staffing levels and staffing models in RACFs [51]. This raises questions about the level of training and the extent to which current training meets the needs of residents, as well as ongoing professional development opportunities and support for nursing assistants. It is also worth commenting that fifty-two countries were identified by study participants as country of birth for those not born in Australia which points to the need for cultural awareness training to be included.

At 13 items, the PANA Skills Questionnaire satisfactorily assesses the skill set of nursing assistants, the largest group of care providers in RACFs [11]. Nursing assistants have previously returned significantly lower total practice (skills) scores compared with other groups of RACF staff such as RNs, ENs, and social workers when bereavement, planning and intervention, and provider coordination were also evaluated using the Palliative Care Survey (PCS) [22]. However, the PCS [22] evaluates skill domains that are not usual practice for nursing assistants. In an effort to address this, items of the PANA_Skills Questionnaire are specific to the role of a nursing assistant. They include the ability to observe changes, communicate and report these, and liaise effectively with other members of the care team, and are essential skills that can directly impact on the quality of residents’ care [52–54]. The inclusion of items specifically related to pain observation and the use of reliable pain assessment tools in the skill set of nursing assistants, are an important implication for the delivery of quality palliative care, with nursing assistants identified in previous studies as an untapped and untrained resource in pain management [55–58].

However, the difficulty of designing an instrument that captures what staff actually do instead of what they think they do or are supposed to do, has been acknowledged [22]. Also, because inexperienced care providers may underestimate the physical and emotional demands and skills required in providing care with a palliative approach [21], they may potentially think they know, for example, how to attend to a dying individual’s care when in fact they do not. Nevertheless, previously described as ‘expert behaviours’ and practices [24, 59], a new language is taking shape around what nursing assistants do, articulated as ‘clinical practice skills’ [17, 60] and, inarguably, the development of nursing assistants’ skills is needed to deliver quality palliative care, and in order to balance the technical, professional and emotional aspects of care [61].

The third major finding is that, when evaluated with the new PANA_Attitudes Questionnaire, a significant difference appears in nursing assistants ‘attitudes relative to their experience in the role. Those participants who had been in the role for more than five years demonstrated significantly more positive attitudes than those with less experience. This result warrants a more focused analysis of attitudes, and of factors known to affect workplace attitudes such as affective state and workplace satisfaction, which may be independent of education and experience [14].

Out of the original 19 attitudinal items, ten are included in the final instrument. When compared with other instruments designed to capture views and attitudes about palliative care [26, 62, 63], the new instrument more specifically reflects the nursing assistants’ role. The items illuminate the concerns and challenges faced by nursing assistants, and identify their unmet needs in education, training and support. For example, feeling comfortable when an individual says they are ready to die was a highly discriminating item in the PANA_Attitudes Questionnaire, indicating a wide variation between participants who feel comfortable when their resident expresses a readiness to die and those who do not. This reflects the findings of Nochomovitz et al. (2010) in their use of the Comfort Scale. The difference is that the Comfort Scale focuses on symptoms, treatment and comfort in talking about death and being present at death, while the new instrument delineates both positive and negative attitudes about providing a palliative approach in the context of the nursing assistants’ role. Making a difference to a person’s day and having an important role in pain assessment and management emphasises and recognises the value of nursing assistants. Overall, the finalised items of the PANA_Attitudes Questionnaire reflect important attitudes required for nursing assistants that are necessary for the provision of psychosocial and spiritual care, acceptance of dying and death, pain management, and managing grief and loss.

The fourth major finding is that there were no significant differences in the interaction between level of education and experience for the attributes of knowledge and attitudes. These results (taken together with the finding that the PANA_Knowledge Questionnaire trended towards a correlation in correct knowledge scores based on experience) support the hypothesis that experience measured as length of time in the nursing assistant’s role is a better indicator of knowledge of a palliative approach than education. In effect, years on the job translate into higher knowledge scores than education. These results derive from a sample in which the majority of participants in Group 1 and Group 2 held the industry recognised qualification (53.9% and 34.1 respectively), and the majority in Group 3 held the higher level Certificate IV in Aged Care^1^ (39.3%) with Certificate III in Aged Care held by 29.7%.

It is understood that education alone does not change palliative care outcomes [64, 65], or practice development, or organisational culture [61]. It does however, play an important part in enhancing knowledge, providing an evidence base for practice and fostering confidence, as well as skill development and competence to enhance clinical practices [60]. Evaluation is part of this process and an important aspect of quality improvement, hence the importance of these tools as a means to identifying the gaps and educational shortfalls in nursing assistants’ knowledge, skills and attitudes. Development of core competencies is important because what has been found to matter most to patients and families across palliative care settings relates to those providing care and how care is provided [66]. Key indicators most associated with patients’ quality of life reported by bereaved families were whether health professionals, including nursing assistants, provided the desired physical comfort and emotional support to the dying person, supported shared decision-making, treated the dying person with respect, attended to the emotional needs of the family, and provided coordinated care [66]. These are all areas identified in the PANA questionnaires.

### Strengths and limitations

A key strength of this study is the inclusion of nursing assistants in developing items, providing role-incumbent knowledge, and enhancing the relevancy of items; and, where possible, in directing the use of terminology. The input of aged care experts from four professional and industry groups increased the validity of the instrument. Pre-testing the instrument was an invaluable component of the development process and allowed the researcher to evaluate the usability of the instrument in the practice setting. There are some limitations to the new instrument and the study sample. First, the sample development and testing took place within greater metropolitan Sydney, an area that does not necessarily reflect regional or rural nursing assistants’ perspectives. The sample is, however, representative of an increasingly culturally diverse and overseas-born workforce that remains predominately female [11, 67]. The questionnaires were developed for an Australian context, although the wider international literature was used to corroborate what nursing assistants know, do and how they feel when providing care with a palliative approach, suggesting uptake in the international setting may be possible. The PANA_Skills Questionnaire measured self-perceived skills and not actual competency against specific criteria. This area of workforce evaluation will be considered for further research. Finally, it is not possible to create an item pool within one manageable instrument that captures every aspect of the construct with subtlety and the complexity of providing palliative care adhering to a defined scope of practice and other factors, such as cultural context. The instrument development process is the first step in a process of ongoing evaluation, review and refinement. Overtime, new items may need to be included to reflect any enhancement to the nursing assistants’ role, and their required knowledge and skills.

## Conclusion

The PANA instruments have demonstrated preliminary evidence for validity and reliability for nursing assistants’ level of education and role responsibility providing a palliative approach in RACFs and, as such, are valid tools by which to identify the educational needs in palliative care of the largest cohort of the aged care workforce.

## Additional files

Additional file 1: Guidelines Framework (DOCX 16 kb)

Additional file 2: The Palliative Approach for Nursing Assistants (PANA) questionnaires URI: http://handle.uws.edu.au:8081/1959.7/566143 (DOCX 28 kb)

## Data Availability

Data sets used in the study analysis are available upon reasonable request from the corresponding author.

## Abbreviations

ACFI: Aged Care Funding Instrument
AIHW: Australian Institute of Health and Welfare
CVI: Content Validity Index
EN: Enrolled Nurse (similar to Licensed Practical Nurse in the USA)
FCM: Facility Care Manager
PANA: Palliative Approach for Nursing Assistants
RACF: Residential Aged Care Facility
RN: Registered nurse
RTO: Registered Training Organisation

## Footnotes

1 Superseded in 2015 by the Certificate in Ageing Support
