# Supplementary material for: An instrument to assess the education needs of nursing assistants within a palliative approach in residential aged care facilities
Source: BMC Palliat Care. 2019 Jul 23;18:61. doi: 10.1186/s12904-019-0447-0 (PMC6647142; doi:10.1186/s12904-019-0447-0)
Supplement: Supplementary file 1 — Guidelines Framework (DOCX 16 kb) [file 12904_2019_447_MOESM1_ESM.docx]

*Guidelines for a Palliative Approach in Residential Aged Care*

| Content | Specific content | Guideline |
| --- | --- | --- |
| 1. A Palliative Approach | When a palliative approach should be implemented | 1-2 |
|  | Where a palliative approach be provided | 3 |
|  | Who provides a palliative approach | 4 |
|  | Dignity and quality of life | 5-6 |
|  | Advance care planning | 7-9 |
|  | Advanced dementia | 10-14 |
| 1. Physical Symptoms: Assessment and Management | Symptom assessment | 15-17 |
|  | Pain management | 18-19 |
|  | Fatigue | 20 |
|  | Nutrition and hydration | 21-26 |
|  | Cachexia | 27-28 |
|  | Dysphagia | 29-30 |
|  | Mouth care | 31-33 |
|  | Skin integrity | 34-35 |
|  | Bowel care | 36-39 |
|  | Dyspnoea | 40-43 |
|  | Complementary therapies | 44-49 |
| 1. Psychological Support | Depression | 50-51 |
|  | Anxiety | 52 |
|  | Delirium | 53 |
|  | Dementia | 54 |
|  | Psychological distress | 55 |
| 1. Family Support |  | 56-59 |
| 1. Social Support – Intimacy and Sexuality | Social support | 60 |
|  | Intimacy | 61 |
|  | Sexuality | 62 |
| 1. Aboriginal and Torres Strait Islander Issues |  | 63-65 |
| 1. Cultural Issues |  | 66-67 |
| 1. Spiritual Support |  | 68-70 |
| 1. Volunteer Support |  | 71-73 |
| 1. End of Life (Terminal Care) |  | 74-75 |
| 1. Bereavement Support |  | 76-78 |
| 1. Management’s Role in  Implementing a Palliative Approach |  | 79 |
